# Borrowing a DNA Repair Enzyme to Fine-Tune Antibody Specificity

**DOI:** 10.1371/journal.pbio.0040403

**Published:** 2006-10-24

**Authors:** Liza Gross

When immunologists discovered in the 1970s that human immune cell receptors recognize tens of millions of antigens—telltale signs of a foreign invader—they were mystified. How could a genome with just 100,000 genes (now estimated at fewer than 25,000) generate this staggering diversity? The answer came with the discovery of a gene rearrangement process that draws on millions of possible permutations to create a unique surface receptor on every T and B lymphocyte—the immune system’s main weapons. But gene diversification of B lymphocytes doesn’t stop there. The immunoglobulin genes, which encode the Y-shaped antibodies that flag infectious microbes for destruction, undergo additional point (single–base pair) mutations that enhance pathogen recognition.

In a new study, Hiroshi Arakawa, Stefan Jentsch, and Jean-Marie Buerstedde find a surprising link between one of these mutation-inducing mechanisms, called immunoglobulin hypermutation, and a DNA repair pathway. When the transcription machinery encounters a DNA lesion, the RAD6 pathway (named after the *Rad6* DNA-repair gene) steps in to either recruit specialized translesion polymerases to bypass the damage, or to copy the correct base sequence from the intact sister strand. While adept at dealing with damaged DNA, the translesion polymerases are “error-prone” compared to standard replicative DNA polymerases. This tendency can be beneficial within the immunoglobulin genes, however, because the introduced mutations can improve antigen recognition of the encoded antibodies. But it was unclear how translesion polymerases are recruited for immunoglobulin hypermutation.

Studies in yeast show that *Rad6* and *Rad18* tag proliferating cell nuclear antigen (PCNA) with a small molecule called ubiquitin. The tag is added to a conserved lysine amino acid residue, called K164—a ubiquitination target in eukaryotes from yeast to humans. Yeast PCNA can undergo modifications at K164 by one ubiquitin tag (mono-ubiquitination), multiple tags (poly-ubiquitination), or by small, ubiquitin-related modifiers (SUMOs) in response to DNA damage. An amino acid substitution from lysine (K) to arginine (R) at position 164 (K164R) in yeast prevents the ubiquitination and SUMOylation but does not compromise the functions of the unmodified PCNA.

The role of PCNA ubiquitination has been well characterized in yeast but not in higher eukaryotes. It had been reported that human PCNA undergoes only mono-ubiquitination at K164, which increases its affinity for two translesion polymerases, Polη and REV1. Working with a chicken B cell line whose genetic tractability has made it a favored model for studying DNA repair and immunoglobulin hypermutation, the authors generated a series of clones carrying the *PCNA^K164R^* mutation either alone or in combination with mutations that inactivated the *RAD18* or *REV1* genes.

**Figure:**
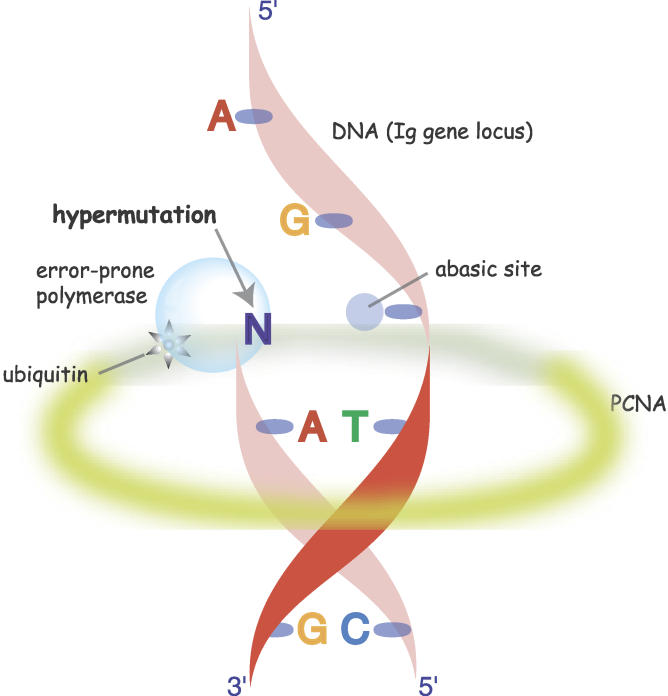
The authors show that vertebrates exploit the PCNA-ubiquitin pathway—a DNA repair pathway—for immunoglobulin hypermutation, most likely through the recruitment of error-prone DNA polymerases.

The authors analyzed extracts from the progenitor line and mutant clones to look for PCNA modifications. Mono-ubiquitinated and “SUMOylated” PCNA was evident in nonmutant cells, but clones carrying the *PCNA^K164R^* mutation showed neither modification. PCNA mono-ubiquitination was markedly reduced, but not eliminated, in cells lacking functional *RAD18* genes. The *PCNA^K164R^* mutation also made cells vulnerable to DNA-damaging agents—likely because PCNA failed to recruit the translesion polymerases—suggesting that vertebrates also require PCNA ubiquitination at the K164 site to survive DNA damage.

All the clones under study expressed immunoglobulin on their surface, allowing the authors to easily track the appearance of harmful mutations in the immunoglobulin variable regions (the targets of hypermutation). Whereas the progenitor line (serving as a control) showed a high rate of immunoglobulin loss (about 35% after 2 weeks culture), the rate was 7-fold reduced in the *PCNA^K164R^* mutant clone. Loss of *RAD18* or *REV1*, however, reduced the rates of mutations by about 2-fold and 3- to 4-fold, respectively.

These results demonstrate that the *PCNA^K164R^* single amino acid substitution not only renders cells sensitive to DNA damage but also dramatically impairs their capacity for immunoglobulin hypermutation. Both effects most likely result from the absence of ubiquitination, since the *RAD18* mutant clone displays a similar but less severe DNA repair and hypermutation defect. Now that it’s clear that the immune system has tailored the ubiquitination-PCNA pathway to its own needs, researchers can begin to work out the molecular details of this appropriation—and perhaps explain how vertebrates managed to maximize the benefits of one pathway by using it in two systems critical for the survival of the organism.

